# Separating the effects of 24-hour urinary chloride and sodium excretion on blood pressure and risk of hypertension: Results from PREVEND

**DOI:** 10.1371/journal.pone.0228490

**Published:** 2020-02-05

**Authors:** Joep van der Leeuw, Martin H. de Borst, Lyanne M. Kieneker, Stephan J. L. Bakker, Ron T. Gansevoort, Maarten B. Rookmaaker

**Affiliations:** 1 Department of Nephrology, University Medical Center Utrecht, Utrecht, The Netherlands; 2 Department of Nephrology, University Medical Center Groningen, Groningen, The Netherlands; International University of Health and Welfare, School of Medicine, JAPAN

## Abstract

**Objective:**

Research into dietary factors associated with hypertension has focused on the sodium component of salt. However, chloride has distinct physiological effects that may surpass the effect of sodium on blood pressure. This study aims to separate the specific effects of chloride and sodium intake on blood pressure.

**Methods:**

We studied 5673 participants from the Prevention of Renal and Vascular End-Stage Disease(PREVEND) study. Urinary chloride(uCl) and sodium(uNa) were measured in two 24-hour collections. We used generalized-linear-regression to evaluate the relation of uCl and uNa with baseline blood pressure and Cox-proportional-hazards-analysis to assess the association with hypertension. Multicollinearity was assessed with Ridge regression.

**Results:**

Baseline 24-hour uCl was 135±39mmol and uNa was 144±54mmol. The correlation between uCl and uNa was high (Pearson’s *r* = 0.96). UCl and uNa had similar non-significant positive and linear associations with blood pressure. In 3515 normotensive patients, 1021 patients developed hypertension during a median follow-up of 7.4 years. UCl and uNa had a comparable but non-significant J-shaped effect on the risk of hypertension. Adding both uCl and uNa to the same model produced instability, demonstrated by Ridge coefficients that converged or changed sign. The single index of uNa minus uCl showed a non-significant higher risk of hypertension of 2% per 10mmol/24-hour difference (HR1.02, 95%CI 0.98–1.06).

**Conclusion:**

UCl and uNa had similar positive but non-significant associations with blood pressure and risk of hypertension and their effects could not be disentangled. Hence, the alleged adverse effects of high salt intake could be due to sodium, chloride or both. This encourages further study into the effect of chloride in order to complement dietary recommendations currently focused on sodium alone.

## Introduction

Hypertension is an important modifiable risk factor for renal and cardiovascular disease[1]. Research into nutritional factors associated with development of hypertension has mainly focused on the cations sodium and potassium[2,3]. Although the importance of chloride in hypertension was suggested more than a century ago, its direct role on blood pressure is still equivocal[4]. As a result, major guidelines contain dietary recommendations focused on sodium alone [5–7].

Despite the focus on sodium, the accompanying anion–chloride–may also play an important role. Chloride is associated with plasma renin suppression, metabolic acidosis and vasoconstriction of the afferent arteriole of the glomerulus and a subsequent reduction in the glomerular filtration rate[8,9]. Preliminary data suggested that the negative effects of high sodium salt consumption may depend on concomitant chloride intake. First, in animals studies the chloride component of dietary salt produced hypertension and suppressed renin release, independent of the associated cation[10–14]. Second, exploratory studies in humans showed that blood pressure was lower with non-chloride sodium salt loading (e.g. sodium bicarbonate, -citrate or -phosphate) compared with sodium chloride loading[15–18]. Third, the beneficial effect of potassium supplementation might be mitigated by concomitant chloride intake[19–21]. Notably, most studies investigating potential benefits of sodium restriction, did not include chloride in their analysis[22,23].

Restricting sodium intake without lowering chloride intake disregard the pathophysiological effects of chloride and may have unintended effects on blood pressure. Since sodium reduction will mostly be effectuated by lowering salt (i.e. sodium chloride) intake, the beneficial effects of sodium reduction in previous studies could also result from concurrent chloride reduction. Although sodium restriction is assumed to result in a similar reduction in chloride, analysis of various food demonstrated that sodium and chloride contents can be different[24,25]. In addition, current dietary recommendations advocate sodium restriction which can be achieved by using sodium depleted salts. Such salts, like as potassium chloride, may contain unrestricted amounts of chloride. We hypothesized that dietary chloride intake has a separate effect on blood pressure that is discernable from the effect of sodium. This could implicate that dietary recommendations should not be limited to sodium alone. In this study, we aim to discern the effects of sodium and chloride intake on blood pressure and the development of hypertension in a large prospective cohort study.

## Materials and methods

The Prevention of Renal and Vascular End-stage Disease (PREVEND) study is a prospective, general population-based cohort study investigating the association of albuminuria with renal and cardiovascular disease. Details of this study are described elsewhere[26]. In total, 8592 individuals (6000 individuals with a urinary albumin concentration of ≥10 mg/ L and 2592 individuals with a urinary albumin concentration <10 mg/L) were recruited between 1997 and 1998 and constitute the PREVEND cohort. For the present analyses, we included participants who took part in the second screening visit, which included an extensive evaluation including urinary chloride measurements. We excluded participants with a history of cardiovascular events (*n* = 410), with missing urinary chloride values (*n* = 134), with an estimated GFR <15 ml/min (*n* = 2) and those without available follow-up data after the second study visit (*n* = 12), leaving 5673 participants for analysis of urinary electrolyte excretion. Urinary sodium and potassium were available for at least one urinary collection for each participant. The association with blood pressure at baseline was evaluated in 4655 participants who did not use antihypertensive drugs. The risk of developing hypertension was evaluated in 3515 patients without hypertension at baseline. The PREVEND study has been approved by the medical ethics committee of the University Medical Center Groningen. Written informed consent was obtained from all participants.

### Data collection

The procedures at each examination in the PREVEND study have been described previously[26]. In brief, each examination included 2 visits to an outpatient clinic separated by 3 weeks. At the first visit, all participants completed a self-administered questionnaire regarding demographics, cardiovascular and renal disease history, smoking habits, alcohol consumption and medication use. Medication use, including antihypertensive drugs, was complemented with information from a pharmacy-dispensing database, which has complete information on the drug use of approximately 95% of subjects in the PREVEND study. Also, fasting blood sample was drawn and stored at −80°C. In the last week before the second visit, subjects had to collect 2 consecutive 24-h specimens after thorough oral and written instruction. During the urine collection, the participants were asked to avoid heavy exercise as much as possible. Subjects were also instructed to postpone the urine collection in case of urinary tract infection, menstruation, or fever. After handing in the urine collections, the urine specimens were stored at -20°C. Blood pressure was assessed during both visits in supine position, every minute for 10 and 8 minutes, respectively, with an automatic Dinamap XL Model 9300 series device (Johnson-Johnson Medical, Tampa, FL). The mean of the last 2 recordings from each visit was used.

### Laboratory measurements and definitions

Determination of urine electrolyte concentration was performed on the 24-h urine specimens by indirect potentiometry with a MEGA clinical chemistry analyzer (Merck, Darmstadt, Germany). Serum and urinary creatinine, serum total cholesterol, triglycerides, and glucose were determined by using Kodak Ektachem dry chemistry (Eastman Kodak, Rochester, NY). Body mass index was calculated as weight in kilograms divided by height in meters squared. Smoking status was categorized as current or never/former and alcohol intake as rarely (≤4 drinks/month), regularly (>4 drinks/month and < = 3 drinks/day) or often (≥4 drinks/day). Education was categorized as low (primary education up to those completing intermediate vocational education), average (higher secondary education), and high (higher vocational education and university). Diabetes mellitus was defined as a fasting plasma glucose ≥7.0 mmol/L or the use of antidiabetic medication. Hypertension was defined as systolic blood pressure (SBP) of ≥140 mmHg, a diastolic blood pressure (DBP) of ≥90 mmHg, or both or the use of antihypertensive agents. Estimated glomerular filtration rate (eGFR) was calculated from the Chronic Kidney Disease Epidemiology Collaboration equation[27].

### Statistical analyses

The urinary chloride, potassium and sodium concentration were multiplied by urine volume to obtain a value in millimoles per 24-hours (24-h). Correlations between 24-h electrolyte concentrations evaluated with Pearson’s correlation coefficient. Second, we evaluated the association between 24-h chloride and sodium excretion and blood pressure at baseline using generalized linear models. Restricted cubic splines were used to examine the optimal functional form of the covariables. Models containing splines were compared with the linear model using conditional likelihood ratio tests. Model assumptions of homoscedasticity and normal distribution of errors were verified graphically by residual versus predicted plots and Q-Q plots. The base models were adjusted for sex and age. Further, fully adjusted models included urinary creatinine, urinary potassium, body mass index, eGFR, current smoking, alcohol consumption, family history of premature cardiovascular disease, and educational attainment. By adjusting for urine creatinine, we adjusted indirectly for both muscle mass and body dimension. We did not adjust for the presence or absence of albuminuria since we considered this covariable as potential intermediary factor in the development of hypertension. Third, the association with development of hypertension was evaluated with multivariable Cox proportional hazards regression. We evaluated the presence of Interaction between uCl and age, sex, albuminuria, C-reactive protein, eGFR, smoking, BMI and family history of premature cardiovascular disease by a likelihood ratio test of the model including a cross-product term. Further, the proportional hazard assumption was verified by correlating scaled Schoenfeld residuals and time and no violations were observed. Since previous analyses demonstrated that the effect of sodium intake on incident hypertension was strongest in patients with albuminuria (defined as ≥15mg/L), we performed a separate analysis in this stratum as sensitivity analysis[2].

In order to detect potentially influential multicollinearity between sodium and chloride in the constructed models we calculated the variance inflation factor (VIF). This parameters indicates how much the variance of a given covariable is increased compared to complete absence of correlation (orthogonality) and is generally agreed to indicate potential model instability if higher than ten[28]. To quantify the effect of multicollinearity on the parameter estimates, we performed Ridge regression with increasing biasing parameter (i.e. penalty factor)[29]. Usually an increasing penalty shrinks coefficients towards zero. Hence, if estimates become larger or change direction, e.g. from negative to positive, they will be considered to be hampered by collinearity[30]. Lastly, we combined urinary sodium and chloride into a single parameter to deal with the problem of multicollinearity.

Data were missing in 2.3% of participants for HDL-cholesterol and triglycerides and <2% for all other variables. Missing data were imputed by single imputation methods using predictive mean matching[31]. Statistical analyses were conducted in R, version 3.4.0 (R Core Team, Vienna, Austria).

## Results

### Baseline characteristics

Patient characteristics are displayed in Table 1. In the entire cohort of 5673 patients, mean baseline 24-h urinary chloride excretion was 135±49 mmol and mean urinary sodium excretion was 145±54 mmol. Of the eligible patients, 4655 patients did not use antihypertensive drugs. Of 3515 participants with normal blood pressure at baseline, 1023 developed hypertension during a median follow-up 7.4 (IQR 3.7–7.6) years. The incidence rate of hypertension was 4.7% per year.

**Table 1:** Baseline characteristics.

Variables		Entire cohort		Without hypertension		Without antihypertensive drugs	
Participants, *n*		5673		3513		4655	
Women, *n* (%)		2941	(52)	1859	(53)	2409	(51)
Age, years		52	±12	49	±10	50	(11)
Race, Caucasian, *n* (%)		5450	(96)	3373	(96)	4475	(96)
Parental history of CVD, *n* (%)		2731	(48)	1630	(46)	2191	(47)
Smoking status, current, *n* (%)		1570	(28)	1022	(29)	1358	(29)
Alcohol consumption, rarely, *n* (%)		2358	(42)	1371	(39)	1842	(39)
BMI, kg/m2		26.6	±4.3	25.7	±3.8	26	(4)
Blood pressure							
	Systolic, mmHg	126	±19	118	±11	123	(17)
	Diastolic, mmHg	73	±9	70	±7	72	(9)
Antihypertensive drugs, *n* (%)		1013	(18)	0	(0)	0	(0)
Total cholesterol, mmol/L		5.4	(4.8–6.1)	5.3	(4.7–6.0)	5.4	(4.7–6.1)
HDL cholesterol, mmol/L		1.2	(1.0–1.4)	1.2	(1.1–1.5)	1.2	(1.1–1.5)
Triglycerides, mmol/L		1.1	(0.8–1.6)	1	(0.8–1.5)	1.1	(0.8–1.5)
Lipid-lowering drugs, *n* (%)		357	(6)	106	(3)	175	(4)
Glucose, mmol/L		4.9	±0.9	4.8	±0.7	4.8	±0.8
Glucose-lowering drugs, *n* (%)		85	(2)	19	(1)	48	(1)
eGFR, mL/min/1.73m2		81	(71–92)	84	(74–94)	83	(73–93)
Urinary excretion							
	Chloride, mmol/24h	135	±50	136	±48	135	±49
	Sodium, mmol/24h	145	±54	145	±53	145	±54
	Potassium, mmol/24h	69	±22	71	±22	70	±22
	Creatinine, mmol/24h	12	±3	13	±3	13	±3
	Albumin/creatinine, mg/mmol	0.7	(0.5–1.3)	0.6	(0.5–1.0)	0.7	(0.5–1.1)

Values are presented as means with standard deviations or medians with interquartile ranges. CVD, premature cardiovascular disease; BMI, body-mass index; HDL, high-density lipoprotein; eGFR, estimated glomerular filtration rate calculated with the Chronic Kidney Disease Epidemiology Collaboration Equation

### Correlation between sodium and chloride excretion

**Fig 1:**
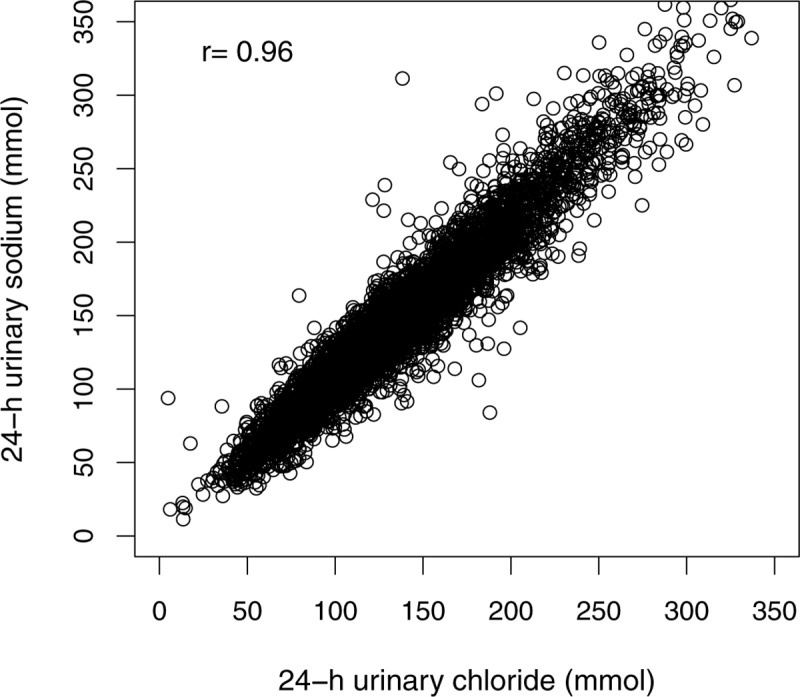
Correlation between urinary sodium and chloride excretion.

In the 5673 participants who attended the second screening visit, there was a very high correlation between 24-h uCl excretion and uNa excretion (Pearson’s *r* = 0.96, *p*<0.001), as illustrated in Fig 1. The mean difference between uNa and uCl was 9.5 mmol [95%CI 21–40]. The correlation between chloride and potassium was much weaker (Pearson’s *r* = 0.52, *p*<0.001).

### Baseline blood pressure

**Fig 2:**
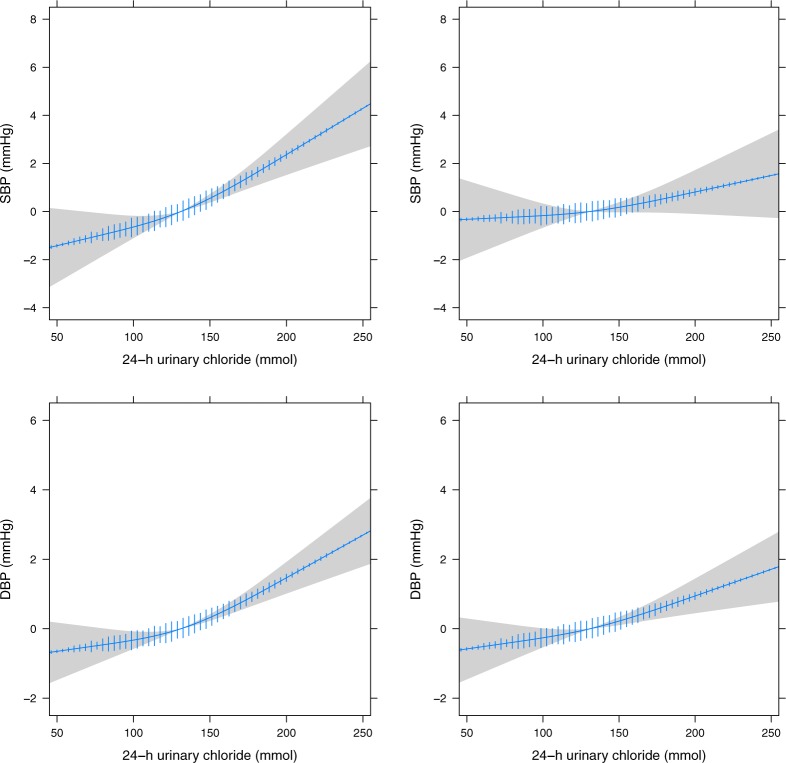
24-h urinary chloride excretion in relation to blood pressure at baseline, with density plot. Adjusted for age and sex (left) and after additional adjustment (right) for age, sex, urinary creatinine, urinary potassium, smoking, body-mass index, history of diabetes, family history of premature cardiovascular disease, educational attainment, alcohol and estimated glomerular filtration rate. SBP, systolic blood pressure; DBP, diastolic blood pressure.

Urinary chloride excretion was nearly linearly related to systolic and diastolic BP in 4655 patients without antihypertensive medication at baseline (Fig 2). In the linear model, each 10 mmol increase in 24-h uCl excretion was associated with a 0.29 mmHg [95%CI 0.19–0.38] increase in systolic and 0.17 mmHg [95%CI 0.12–0.22] in diastolic BP. The second model accounted for potential confounders and attenuated the associations. After adjustment, an increase of 10 mmol in 24-h uCl was associated with a non-significant 0.09 mmHg [95%CI -0.02–0.20] higher systolic BP and a significant 0.12 mmHg [95%CI 0.06–0.18] higher diastolic BP. 24-h UNa excretion had a similar non-significant association with BP (S1 Fig). Analysis of interaction by albuminuria as a continuous variable showed smaller increases in systolic BP with each increment in uCl at higher values of urinary albumin (*p* for interaction <0.001). When specifying tertiles of albuminuria, an increased effect for uCl was observed in the middle tertile but a decreased effect in the highest tertile. Higher age increased the effect of uCl on systolic BP whereas it decreased the effect on diastolic BP (*p* for interaction <0.001).

The model combining both uCl and uNa was hampered by high collinearity (VIF = 12) resulting in opposite associations for chloride and sodium with systolic and diastolic BP (S2 Fig). When applying Ridge regression, the addition of a small penalty factor resulted in the coefficients of uCl and uNa to converge. The coefficient of uCl decreased whereas the coefficient of uNa increased with higher biasing parameter, indicating that the model could not reliably separate the effect of chloride and sodium.

### Development of hypertension

**Fig 3:**
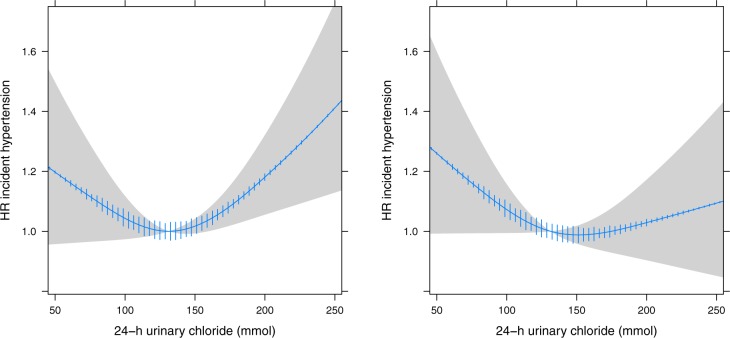
Urinary chloride excretion and the risk of hypertension. Adjusted for age and sex (left) and fully adjusted model (right) for urinary creatinine, urinary potassium, smoking, body-mass index, history of diabetes, family history of premature cardiovascular disease, educational attainment, alcohol and estimated glomerular filtration rate. HR, hazard ratio.

In 3513 patients without hypertension at baseline, uCl excretion showed a J-shaped association with the risk of hypertension (Fig 3). The deviation from linearity was significant (*p*<0.01); therefore uCl was modelled as a spline. The spline of uCl was significant in the base model (*p* = 0.01) but not in the full model (*p* = 0.16). The risk of hypertension was lowest at a uCl level of 151 mmol in the fully adjusted model. The constructed tertiles of uCl excretion showed a trend towards an increased risk of hypertension in both the lowest and highest tertile (Table 2). No significant interactions were observed. The association of uNa with the risk of hypertension was non-significant and similar to uCl (S3 Fig). Sensitivity analyses performed in patients with a albuminuria showed a stronger association for both chloride and sodium with risk of hypertension (*p* = 0.07 for chloride and *p* = 0.02 for sodium after full adjustment, Table 2).

**Table 2:** Association between chloride excretion per 24-h in tertiles and risk of hypertension. Separately shown in overall population (n = 3513) and patients with albuminuria (>15mg/24h, n = 638).

	1st tertile	2nd tertile	3rd tertile
* *	<111 mmol/24h	111–151 mmol/24h	>151 mmol/24h
**Overall population**			
No. of cases	344	311	368
Age and sex-adjusted HR	1.16 (0.99–1.35)	1.0 (reference)	1.20 (1.03–1.40)
Multivariable adjusted HR^a^	1.19 (1.02–1.40)	1.0 (reference)	1.06 (0.91–1.25)
**Patients with albuminuria**			
No. of cases	75	82	137
Age and sex-adjusted HR	1.30 (0.95–1.79)	1.0 (reference)	1.29 (0.98–1.70)
Multivariable adjusted HR^a^	1.39 (0.99–1.95)	1.0 (reference)	1.22 (0.91–1.63)

Hazard ratio's derived from Cox proportional-hazards model with chloride as independent variable in three categories based on tertiles of 24-h chloride excretion. The 2nd tertile serves as reference category.

^a^ Additionally adjusted for smoking, BMI, diabetes, TC/HDL ratio, urinary potassium, urinary creatinine, log-transformed eGFR, alcohol consumption, family history of premature CVD and educational attainment.

**Fig 4:**
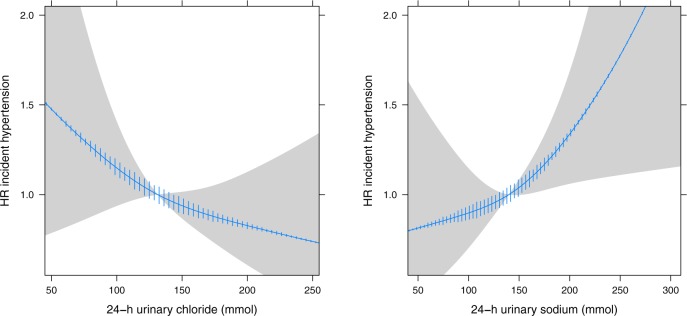
**Urinary chloride (left) and sodium (right) excretion and the risk of hypertension incorporated in the same model.** Age and sex adjusted. HR, hazard ratio.

**Fig 5:**
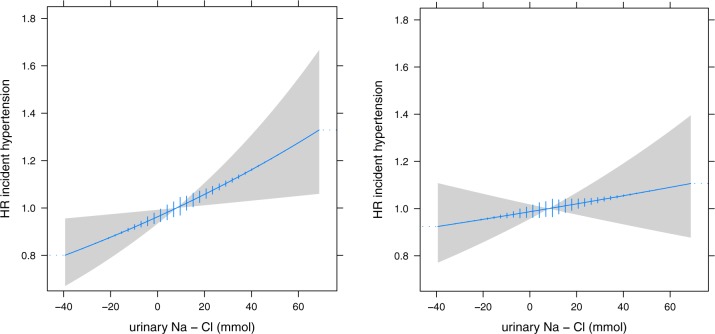
**Difference in urinary sodium and chloride excretion and the risk of hypertension (left) and after further adjustment (right).** Adjusted for urinary creatinine, urinary potassium, smoking, body-mass index, history of diabetes, family history of premature cardiovascular disease, educational attainment, alcohol and estimated glomerular filtration rate (right). HR, hazard ratio.

Combining uCl and uNa in the same model resulted in an inverse association of uCl with the risk of hypertension (Fig 4), probably because of overlapping information (VIF = 11). In the Ridge regression model the initially negative coefficient of uCl became positive while the coefficient for uNa remained positive with increasing penalty factor, demonstrating the impact of multicollinearity. Next, we combined uCl and uNa into a single index (Fig 5). The difference between uNa and uCl showed a linear relation with the risk of hypertension. Each 10 mmol increment between uNa and uCl excretion (indicating a higher sodium than chloride intake) resulted in additional risk of 5% (HR 1.05, 95%CI 1.01–1.09), which disappeared after adjustment (HR1.02, 95%CI 0.98–1.06). Additional analysis with the uNa to uCl ratio showed a similar pattern with a non-significant increased risk of incident hypertension for higher ratios indicating more sodium compared to chloride intake (S4 Fig). For both combined indices, further adjustment for the urinary sodium to potassium ratio did not change the slope of associations.

## Discussion

The cation sodium is often used interchangeably for salt (i.e. sodium chloride) and is the alleged culprit of the adverse effects on blood pressure and cardiovascular disease. The most abundant accompanying anion, chloride, has received little attention but could have a pivotal role. In this study we showed the difficulty of disentangling the effects of sodium and chloride because sodium and chloride urinary excretions were highly correlated. Nonetheless, differences in sodium versus chloride excretions occurred at an individual level. Higher 24-h uCl excretion was positively but not significantly associated with baseline BP and development of hypertension similar to and inseparable from the effect of higher uNa excretion. Therefore, we argue that the role of chloride cannot be discarded and should be considered in clinical studies and dietary recommendations on salt intake.

In this observational cohort study, we tried to separate the collinear effects of both ions with different analytical methods. The negative effects of multicollinearity in statistical models primarily pertain to inadequate estimates of uncertainty such as confidence intervals and *p*-values[28]. Further, disentangling the independent contribution of the correlated covariables poses a problem when they contain overlapping information, like sodium and chloride. Including both variables in one statistical model can yield effect estimates of implausible magnitude or estimates in opposed direction of what was expected[30]. Potential solutions are removing either variable from the model, using Ridge regression, combining the two covariables into a single index or using principal component analysis[28,29]. In the present studies we applied the first three approaches. First, we evaluated chloride and sodium excretion separately and showed similar associations with BP and risk of hypertension for each ion. Next, we applied Ridge regression, which increases the variance relative to the covariance in order to limit the effect of multicollinearity[29]. However, the estimate produced by the Ridge model are biased and can only be used to get an impression of how much the model is affected by multicollinearity. In this study, the introduction of a small penalty factor in the cross-sectional analyses yielded models with coefficients for sodium and chloride which converged to each other. This convergence indicates that both variables contain similar information. In the prospective analysis, the coefficient for chloride changed sign (i.e. became positive) after applying a penalty factor, again illustrating the effect of multicollinearity. Lastly, we combined sodium and chloride into a single variable both as their difference and ratio. Both indices showed that a higher sodium compared to chloride intake might have an adverse effect on the risk of hypertension, although the association was non-significant. The lower sodium to chloride ratio could also indicate a higher intake of other cations, notably potassium, which has a blood pressure lowering effect[32]. Nonetheless, the adjusted models incorporated potassium or the sodium to potassium ratio and showed a weaker, but still positive association. Further, in the cross-sectional analysis a steeper association of 24-h chloride excretion with BP was observed at higher levels of chloride intake very similar to the stronger effects reported at higher intake of sodium in other studies[33]. In the prospective analysis, we observed a J-shaped association for the risk of developing hypertension. Such an association is also observed in studies evaluation sodium excretion and risk of cardiovascular events or death[34,35]. A possible explanation is reverse causation, i.e. people reduce sodium and chloride intake due to illness or health advice. We excluded participants with previous cardiovascular disease and studied a general population sample, which is considered to result in a lower risk of reverse causation[36].Nonetheless, the fact that the risk associated with low 24-h uCl was higher in patients with albuminuria who are more likely to have had dietary counseling, might indicate some remaining reverse causation.

The focus of current dietary recommendations on sodium follows from the selective evaluation of cations in dietary studies thus far. The present study is the first to attempt to separate the effects of the cation and anion and showed their high correlation and comparable effects on blood pressure. The current recommendations on selective sodium restriction are based on the large observational studies such as INTERSALT, INTERMAP and PURE[33,37–39]. Further, meta-analyses of more than 30 trials confirmed that reducing sodium intake lowers BP[23,40]. Although these studies measured a range of cations, creatinine and albumin, they did not evaluate anions. The interventions to reduce sodium intake consisted mainly of dietary advice or provision of key foods with lower sodium content, both of which are likely to have reduced chloride intake as well. However, sodium and chloride are not completely tied together. Although sodium is mostly consumed as sodium chloride in a typical Western diet, they do not necessarily go hand in hand[24]. In more than thousand food items, the sodium chloride content was calculated by measuring the sodium and chloride ion separately and converting the measured ion concentrations into the corresponding salt. The estimated salt content was different for seven out of ten foods. The differences ranged up to a 43% higher salt content for specific food items for the sodium-based approach. This implies that sodium and chloride may originate from different natural sources or food additives. Further, food composition and main sources of sodium and chloride differ by cultural context and dietary habits around the world[41]. In addition, specific dietary recommendations like the use of sodium-depleted salts (e.g. potassium chloride) could result in isolated higher chloride intake. In studies evaluating potassium chloride supplementation, the beneficial effect on BP could be due to the introduction of potassium irrespective of the potential adverse effect of sodium or chloride[42]. In conclusion, our results agree with previous observational studies on the effect of sodium intake on BP by demonstrating a similar positive association. However, thus far sodium was used as proxy for salt and the effect of chloride was not evaluated. Although sodium restricting is likely to lower chloride intake, food composition and salt replacements can indeed vary with respect to ion content. Therefore, the findings from this study encourage reevaluation of the dietary salt recommendations based on sodium only.

To our knowledge this is the first study to quantify the correlation between sodium and chloride excretion and their association with BP and risk of hypertension in a large prospective Western cohort. Strengths of the current study include the large number of participants, the long follow-up time and the availability of two 24-h urine collections per individual to reliably predict electrolyte intake[43]. In addition, we used different statistical approaches to evaluate the impact of multicollinearity and demonstrated the inability to clearly separate the effects of chloride and sodium. The combination of both ions into a single index offered additional insight, but only shed light on the ratio of the two ions instead of their individual contribution. The observational nature of this study was the major limitation and prevented us from separating the effects of sodium and chloride. Further, in the present analyses the associations between uCl and uNa and incident hypertension were of similar direction but no longer significant after full adjustment, in contrast to previous findings. This can be explained by the two thousand fewer available participants without hypertension at the second screening visit when uCl was measured, as well as by previous reports showing the most pronounced associations in patients with hypertension or markers of endothelial dysfunction[2,33]. We explored whether the effect of chloride was modified by vascular endothelial dysfunction defined by albuminuria and observed a stronger effect of uCl on development of hypertension with higher urinary albumin excretion in concurrence with previous work[2]. Lastly, higher age attenuated the effect of uCl on baseline systolic BP while at the same increasing the effect on diastolic BP, which might indicate differential effect of salt loading on BP in older people[39].

In conclusion, the adverse effects of chloride and sodium measured by 24-h urinary excretion were difficult to disentangle due to their high correlation and comparable non-significant effects on blood pressure and risk of hypertension. Hence, the alleged adverse effects of a high salt intake could be due to sodium, chloride or both. An intervention study with different combinations of cations and anions would be required in order to understand their relative contribution to the development of hypertension. For now, guidelines committees should consider formulating sodium chloride targets rather than sodium targets alone.

## Supporting information

S1 Fig 24-h urinary sodium excretion in relation to blood pressure at baseline. Adjusted for age and sex, with density plot. SBP, systolic blood pressure; DBP, diastolic blood pressure.
(PDF)

S2 Fig Urinary chloride and sodium when combined together in an age and sex-adjusted model. Related to baseline systolic blood pressure (SBP; upper panels) and diastolic blood pressure (DBP; lower panels), with density plots.
(PDF)

S3 Fig 24-h urinary sodium excretion and the risk of hypertension. With base model (left) and fully adjusted model for urinary creatinine, urinary potassium, smoking, body-mass index, history of diabetes, family history of premature cardiovascular disease, educational attainment, alcohol and estimated glomerular filtration rate (right). HR; hazard ratio.
(PDF)

S4 Fig Ratio of 24-h urinary sodium to chloride excretion and the risk of hypertension. Base model (left) and after further adjustment for covariables (right). HR; hazard ratio.
(PDF)
