# Supplementary material for: Separating the effects of 24-hour urinary chloride and sodium excretion on blood pressure and risk of hypertension: Results from PREVEND
Source: PLoS One. 2020 Feb 5;15(2):e0228490. doi: 10.1371/journal.pone.0228490 (PMC7001936; doi:10.1371/journal.pone.0228490)

S1 Fig. 24-h urinary sodium excretion in relation to blood pressure at baseline. Adjusted for age and sex, with density plot. SBP, systolic blood pressure; DBP, diastolic blood pressure.

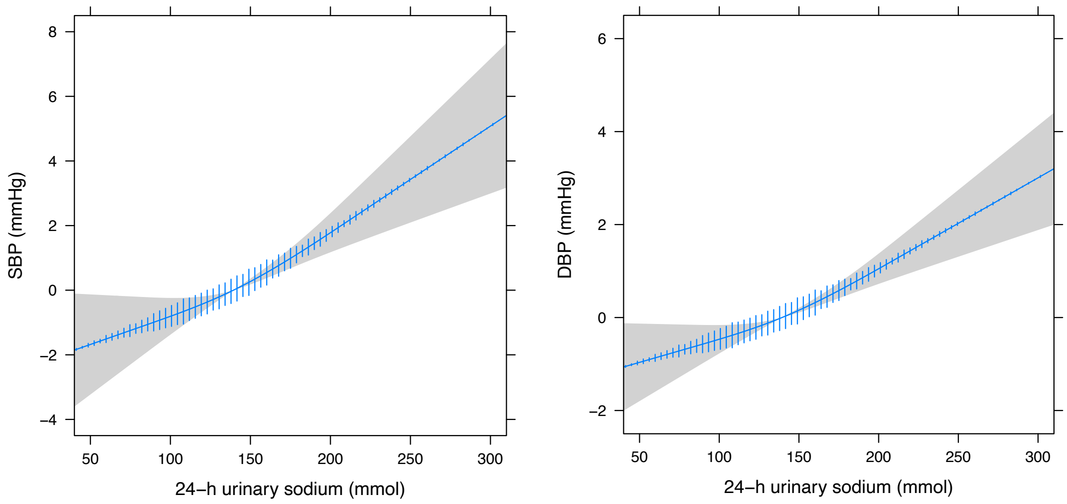

Supplement: S1 Fig — Adjusted for age and sex, with density plot. SBP, systolic blood pressure; DBP, diastolic blood pressure. (PDF) [file pone.0228490.s001.pdf]
