# Supplementary material for: Separating the effects of 24-hour urinary chloride and sodium excretion on blood pressure and risk of hypertension: Results from PREVEND
Source: PLoS One. 2020 Feb 5;15(2):e0228490. doi: 10.1371/journal.pone.0228490 (PMC7001936; doi:10.1371/journal.pone.0228490)

**S2 Fig. Urinary chloride and sodium when combined together in an age and sex-adjusted model.**

Related to baseline systolic blood pressure (SBP; upper panels) and diastolic blood pressure (DBP; lower panels), with density plots.

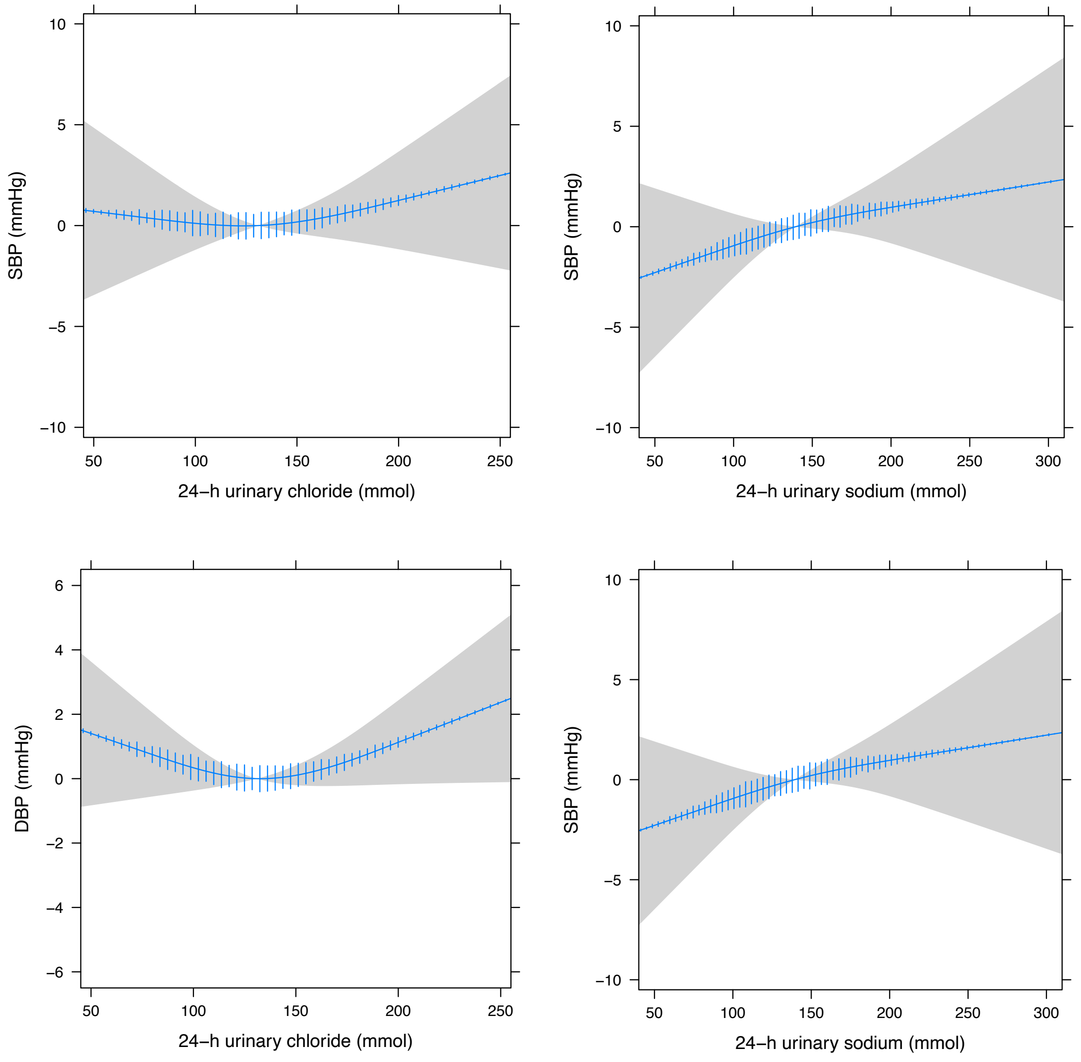

Supplement: S2 Fig — Related to baseline systolic blood pressure (SBP; upper panels) and diastolic blood pressure (DBP; lower panels), with density plots. (PDF) [file pone.0228490.s002.pdf]
