# Supplementary material for: Separating the effects of 24-hour urinary chloride and sodium excretion on blood pressure and risk of hypertension: Results from PREVEND
Source: PLoS One. 2020 Feb 5;15(2):e0228490. doi: 10.1371/journal.pone.0228490 (PMC7001936; doi:10.1371/journal.pone.0228490)

S3 Fig. 24-h urinary sodium excretion and the risk of hypertension. With base model (left) and fully adjusted model for urinary creatinine, urinary potassium, smoking, body-mass index, history of diabetes, family history of premature cardiovascular disease, educational attainment, alcohol and estimated glomerular filtration rate (right). HR; hazard ratio.

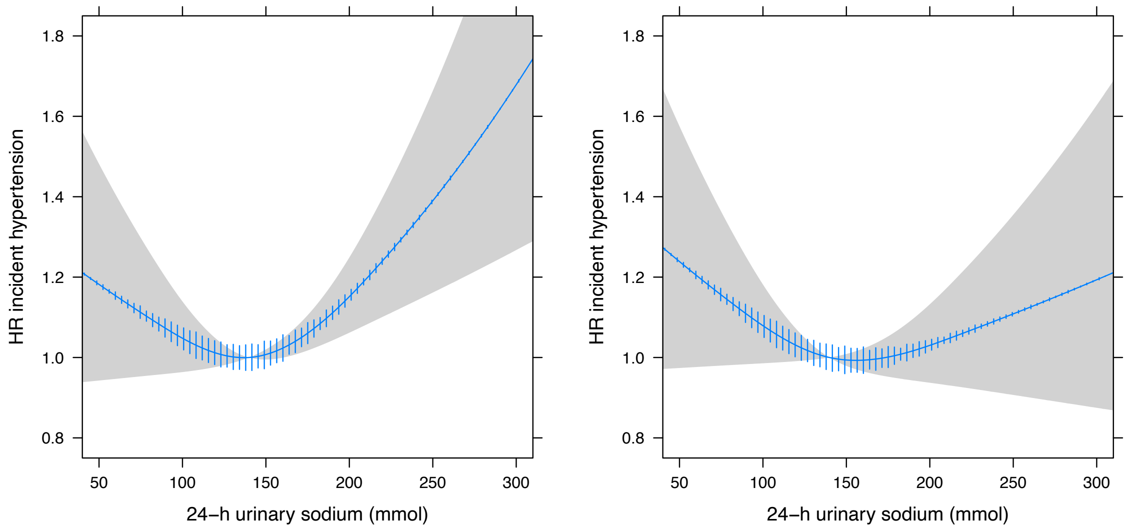

Supplement: S3 Fig — With base model (left) and fully adjusted model for urinary creatinine, urinary potassium, smoking, body-mass index, history of diabetes, family history of premature cardiovascular disease, educational attainment, alcohol and estimated glomerular filtration rate (right). HR; hazard ratio. (PDF) [file pone.0228490.s003.pdf]
