# Supplementary material for: Separating the effects of 24-hour urinary chloride and sodium excretion on blood pressure and risk of hypertension: Results from PREVEND
Source: PLoS One. 2020 Feb 5;15(2):e0228490. doi: 10.1371/journal.pone.0228490 (PMC7001936; doi:10.1371/journal.pone.0228490)

S4 Fig. Ratio of 24-h urinary sodium to chloride excretion and the risk of hypertension. Base model (left) and fully adjustment for covariables (right). HR; hazard ratio.

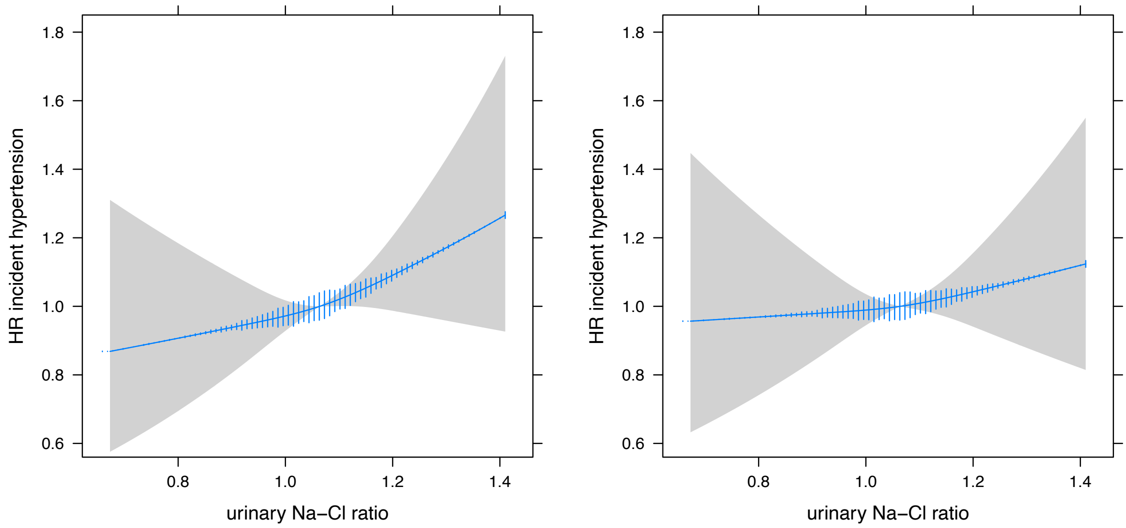

Supplement: S4 Fig — Base model (left) and after further adjustment for covariables (right). HR; hazard ratio. (PDF) [file pone.0228490.s004.pdf]
